# Iron Status and the Acute Post-Exercise Hepcidin Response in Athletes

**DOI:** 10.1371/journal.pone.0093002

**Published:** 2014-03-25

**Authors:** Peter Peeling, Marc Sim, Claire E. Badenhorst, Brian Dawson, Andrew D. Govus, Chris R. Abbiss, Dorine W. Swinkels, Debbie Trinder

**Affiliations:** 1 School of Sport Science, Exercise and Health, The University of Western Australia, Crawley, Western Australia, Australia; 2 Centre for Exercise and Sports Science Research, School of Exercise and Health Science, Edith Cowan University, Western Australia, Australia; 3 Department of Laboratory Medicine, Laboratory of Genetic, Endocrine and Metabolic diseases, Radboud University Medical Centre, Nijmegen, The Netherlands; 4 Hepcidinanalysis.com, Nijmegen, The Netherlands; 5 School of Medicine and Pharmacology, The University of Western Australia, Fremantle, Western Australia, Australia; Lady Davis Institute for Medical Research/McGill University, Canada

## Abstract

This study explored the relationship between serum ferritin and hepcidin in athletes. Baseline serum ferritin levels of 54 athletes from the control trial of five investigations conducted in our laboratory were considered; athletes were grouped according to values <30 μg/L (SF<30), 30–50 μg/L (SF30–50), 50–100 μg/L (SF50–100), or >100 μg/L (SF>100). Data pooling resulted in each athlete completing one of five running sessions: (1) 8×3 min at 85% vVO_2peak_; (2) 5×4 min at 90% vVO_2peak_; (3) 90 min continuous at 75% vVO_2peak_; (4) 40 min continuous at 75% vVO_2peak_; (5) 40 min continuous at 65% vVO_2peak_. Athletes from each running session were represented amongst all four groups; hence, the mean exercise duration and intensity were not different (p>0.05). Venous blood samples were collected pre-, post- and 3 h post-exercise, and were analysed for serum ferritin, iron, interleukin-6 (IL-6) and hepcidin-25. Baseline and post-exercise serum ferritin levels were different between groups (p<0.05). There were no group differences for pre- or post-exercise serum iron or IL-6 (p>0.05). Post-exercise IL-6 was significantly elevated compared to baseline within each group (p<0.05). Pre- and 3 h post-exercise hepcidin-25 was sequentially greater as the groups baseline serum ferritin levels increased (p<0.05). However, post-exercise hepcidin levels were only significantly elevated in three groups (SF30–50, SF50–100, and SF>100; p<0.05). An athlete's iron stores may dictate the baseline hepcidin levels and the magnitude of post-exercise hepcidin response. Low iron stores suppressed post-exercise hepcidin, seemingly overriding any inflammatory-driven increases.

## Introduction

Recently, the mechanisms relating to iron deficiency in athletes has been increasingly investigated, with a shift in focus from the more traditionally accepted avenues of exercise-induced iron loss such as hemolysis, sweating and gastrointestinal bleeding (for review see [1]), to the influence of the iron regulatory hormone known as hepcidin [2]–[4]. Recently, factors that may affect the activity of this hormone such as training frequency, exercise modality and nutritional practices have been established [5]–[7]. Hepcidin is a liver produced peptide, which acts to regulate iron absorption from the intestine and recycling from the macrophage via its interaction (internalisation and degradation) with the body's only known cellular iron exporter, ferroportin [8]. Increases in hepcidin levels usually occur as a homeostatic response to inflammatory stimuli (namely the inflammatory cytokine interleukin-6: IL-6) or elevated iron levels [9], ultimately reducing dietary iron absorption from the small intestine, and reducing the ability of macrophages to recycle iron from senescent erythrocytes [10]. However, research investigating the time-course of exercise-induced hepcidin response shows that the levels of this hormone seem to peak some 3–6 h subsequent to the peak in IL-6 elevation after an exercise bout [3]. No doubt, such timing is synonymous with that at which an athlete may be consuming meals that contain the majority of their dietary iron intake, and as such, it has been suggested that elevated post-exercise hepcidin levels may contribute to the high number of athletes commonly diagnosed with iron deficiency [11].

Nevertheless, evidence from our laboratory initially proposed that the hepcidin response of an athlete with compromised iron levels may be different to that of an athlete with healthy iron stores [3]. However, this paper assessed hepcidin in urine rather than serum, and only showed three athletes presenting as non-hepcidin responders in the post-exercise period; hence, statistical analysis on this data became difficult. More recently, it was shown that 9 weeks of basic combat training resulted in reductions to the serum ferritin levels of military personnel, without impacting on baseline hepcidin levels [12]. However, these authors also reported that the baseline hepcidin levels of soldiers presenting with compromised iron stores were lower than those who presented with healthy serum ferritin levels [12]. Subsequently, Auersperger et al., [13] also showed a decline in serum ferritin and baseline hepcidin levels within their group, after the completion of an 8 week exercise-training intervention. Regardless, neither of these two investigations [12], [13] quantified the hepcidin response in the acute post-exercise period, when both low serum ferritin levels and exercise-induced inflammation are present.

The prospect of an altered post-exercise hepcidin response in iron deplete athletes is not obscure, since the inverse homeostatic function of the body is to reduce the circulating levels of hepcidin in response to low iron levels, hypoxia, or when iron is needed for processes such as erythropoiesis [14]. For example, it has been shown that the hepcidin response to a prolonged hypoxic exposure is attenuated [15], possibly due to the impact of erythropoietin (EPO) stimulation on the bone marrow to produce new red blood cells [16], which requires the utilisation of the body's iron stores. However, it has also been suggested that EPO, hypoxia inducible factors, or growth differentiation factors, all of which are increased during hypoxia, may directly effect hepcidin expression [16], [17]. Regardless, despite the proposition that the hepcidin response of an iron-deplete athlete may be different to that of an athlete with healthy iron stores, the exercise-induced inflammatory response will still be evident in such athletes, posing the question of which regulatory process (inflammation or low iron levels) will have the over-riding influence on the subsequent post-exercise hepcidin response.

As a result, it was the aim of this investigation to determine the influence of an exercise stimulus on the acute post-exercise hepcidin response of athletes characterised as iron deplete (serum ferritin <30 μg/L), and to compare this response to a group of athletes with sub-optimal (serum ferritin 30–50 μg/L), healthy (serum ferritin 50–100 μg/L), and high (serum ferritin >100 μg/L) iron stores.

## Methods

### Ethics Statement

This study utilised pooled data from five separate investigations conducted in our laboratory. Each investigation individually received approval from the Human Ethics Committee of the University of Western Australia conforming to the Declaration of Helsinki on the use of human subjects. Written informed consent was obtained from all participants prior to their participation in each investigation. Only data from the control trial of each investigation was used here. As a result, the data set is not impacted by any intervention other than exercise.

### Experimental Overview

The data of 54 trained runners or triathletes (38 males and 16 females) are represented below (sample population mean (± standard deviation) for age: 25.8 (±6.6) years; body mass: 67.1 (±10.1) kg; stature: 174.2 (±8.4) cm; and VO_2peak_: 60.1 (±7.3) ml/kg/min). All athletes presented as medically fit, with no underlying health concerns. From this cohort, baseline serum ferritin levels were considered, and the athletes were categorised into four groups;

1. Serum ferritin <30 μg/L (SF<30): n = 12 (4 male and 8 female)
2. Serum ferritin 30–50 μg/L (SF30–50): n = 8 (3 male and 5 female)
3. Serum ferritin 50–100 μg/L (SF50–100): n = 14 (13 male and 1 female)
4. Serum ferritin >100 μg/L (SF>100): n = 20 (18 male and 2 female)

It should be noted that a variety of cut-off values appear in the literature for the determination of an iron deficiency in athletes [1], [2], [12], [18]. However, the upper limit of 30 μg/L for the SF<30 group in the current investigation is based on the standards for determination of an iron deficiency as directed by the Royal College of Pathologist Australasia [19]. Additionally, we have distinguished a group of athletes that would be considered as presenting with ‘sub-optimal’ iron status in an elite sport institute setting, whereby the iron status of such an athlete would be monitored over time. Garvican et al., [18] have described this as ‘sub-optimal’ ferritin levels, and recently used the upper limit cut-off classification as 65 μg/L. Regardless, a more conservative upper limit of 50 μg/L was used here to define this group. Thereafter, based on the suggestions of Garvican et al., [18], the final two groups were determined as athletes presenting with healthy serum ferritin levels (50–100 μg/L), and those which would be considered high in an athletic population (>100 μg/L).

Due to data pooling, it must be highlighted that the exercise stimulus was varied between each investigation, such that there are five different running sessions that participants may have completed. However, due to standardised pre-testing protocols from our laboratory, all participants were instructed not to exercise during the 24 h pre-testing period, and a routine diet for each individual throughout this time was encouraged. Furthermore, all running sessions were completed in the morning, thereby avoiding any potential circadian influence. In all five investigations, the participants initially completed a running-based graded exercise test (GXT), as per the method consistently used and previously reported by our laboratory [4]. The GXT was used for the determination of peak oxygen consumption (VO_2peak_) and the associated running velocity (vVO_2peak_). Subsequently, the participants then completed one of the following five running sessions on a motorised treadmill in the laboratory:

- Interval Run 1 (INT_1_; n = 10): A 5 min warm-up at 60% vVO_2peak_, followed by 8×3 min repeats at 85% vVO_2peak_ interspersed with 90 s of recovery between repetitions.
- Interval Run 2 (INT_2_; n = 12): A 5 min warm-up at 65% vVO_2peak_, followed by 5×4 min repeats at 90% vVO_2peak_ interspersed with 90 s of recovery between repetitions.
- Long Slow Distance 1 (LSD_1_; n = 12): A 5 min warm-up at 60% vVO_2peak_, followed by a continuous 90 min run at 75% vVO_2peak_.
- Long Slow Distance 2 (LSD_2_; n = 10): A 5 min warm-up at 60% vVO_2peak_, followed by a continuous 40 min run at 75% vVO_2peak_.
- Long Slow Distance 3 (LSD_3_; n = 10): A 5 min warm-up at 60% vVO_2peak_, followed by a continuous 40 min run at 65% vVO_2peak_.

During each of the above trials, heart rate (HR) was collected as a measure of exercise intensity (Polar, Finland), which has been compared and presented with exercise duration data below. Although the exercise stimulus was varied between investigations, the spread of athletes amongst the four classifications of iron status was such that each running session and gender is represented across the four groups. As a result, the mean exercise duration and intensity of each group were similar (data presented below).

Blood collection protocols used here were consistent between each of the five aforementioned investigations. Venous blood samples were collected at three separate time points (pre-exercise, post-exercise, and 3 h post-exercise) using a 21-gauge needle into an 8.5 mL SST Gel separator tube. Serum samples were allowed to clot for 60 min at room temperature, before subsequently being centrifuged at 10°C and 3000 rpm for 10 min. Serum supernatant was divided into 1 mL duplicate aliquots and stored at −80°C until further analysis. Frozen serum samples were taken to the Royal Perth Hospital pathology laboratory to be analysed for circulating levels of serum ferritin, serum iron, and IL-6 (pre- and post-exercise). Furthermore, duplicate samples were sent to Radboud University Medical Centre (Nijmegen, The Netherlands) for serum hepcidin-25 analysis (pre- and 3 h post-exercise). The specified time points for the analysis of each blood variable were chosen to reflect their peak post-exercise activity from prior research [3]. Details pertaining to the methods and laboratory-specific precision of these assays have been previously reported by our group, and are consistent across our investigations [6].

### Statistical Analysis

Data are presented as mean and standard deviation (±SD), unless otherwise stated. A series of one-way ANOVA for independent samples were used to compare between group differences for measures of exercise duration and intensity, serum ferritin, serum iron, IL-6, and hepcidin responses. In the event of a main effect, least significant difference post-hoc pairwise comparisons were made. Additionally, Pearson's correlation coefficients were calculated to describe any associations between blood parameters. The alpha level was accepted at p≤0.05.

## Results

As a result of the spread of athletes from each running session across the four groups, the mean running duration was 49±14 min (SF<30); 51±18 min (SF30–50); 57±22 min (SF50–100); and 63±24 min (SF>100), with no significant differences between groups (p = 0.30). Similarly, for running intensity, the mean HR was 170±11 bpm (SF<30); 163±15 bpm (SF30–50); 167±14 (SF50–100); and 169±11 bpm (SF>100), also with no differences between groups (p = 0.62).

The mean baseline and post-exercise serum ferritin and serum iron levels are presented in Table 1. Significant between group differences were evident for baseline (p = 0.01) and post-exercise (p = 0.01) serum ferritin levels; however, there were no between group differences for serum iron levels at either time point (p = 0.82 and p = 0.71, respectively). The within group analysis showed that serum ferritin was significantly elevated post-exercise in all groups except SF<30 (SF<30: p = 0.06; SF30–50: p = 0.04; SF50–100: p = 0.01; SF>100: p = 0.01); and that serum iron was significantly increased in all groups except SF30–50 (SF<30: p = 0.01; SF30–50: p = 0.10; SF50–100: p = 0.01; SF>100: p = 0.01).

**Table 1:** Mean (±SD) baseline and post-exercise serum ferritin, serum iron, and Interleukin-6 levels in athletes grouped by serum ferritin <30 μg/L (SF<30), between 30–50 μg/L (SF30–50), between 50–100 μg/L (SF50–100), and >100 μg/L (SF>100).

		Serum Ferritin		Serum Iron		Interleukin-6	
		*Baseline*	*Post-Exercise*	*Baseline*	*Post-Exercise*	*Baseline*	*Post-Exercise*
**Serum Ferritin**	*mean*	19.0	20.1	16.2	18.1†	1.3	3.8†
**<30**	*SD*	5.5	5.0	7.0	7.0	2.4	6.1
**Serum Ferritin**	*mean*	43.1	45.9†	19.3	20.5	1.1	3.5†
**30–50**	*SD*	5.6	6.8	9.9	9.4	0.3	2.6
**Serum Ferritin**	*mean*	69.9	75.5†	17.8	20.1†	1.7	4.3†
**50–100**	*SD*	13.5	17.7	8.4	8.2	2.1	2.8
**Serum Ferritin**	*mean*	152.5	165.3†	18.0	21.3†	1.1	5.6†
**>100**	*SD*	56.6	62.1	4.9	5.9	1.0	6.7

† Significantly greater than pre-exercise.

The pre- and post-exercise IL-6 levels are presented in Table 1. No significant between group differences were found at either pre- (p = 0.49) or post-exercise (p = 0.73). However, the post-exercise IL-6 levels were significantly elevated compared to baseline within each group (SF<30: p = 0.04; SF30–50: p = 0.02; SF50–100: p = 0.01; SF>100: p = 0.01).

**Figure 1:**
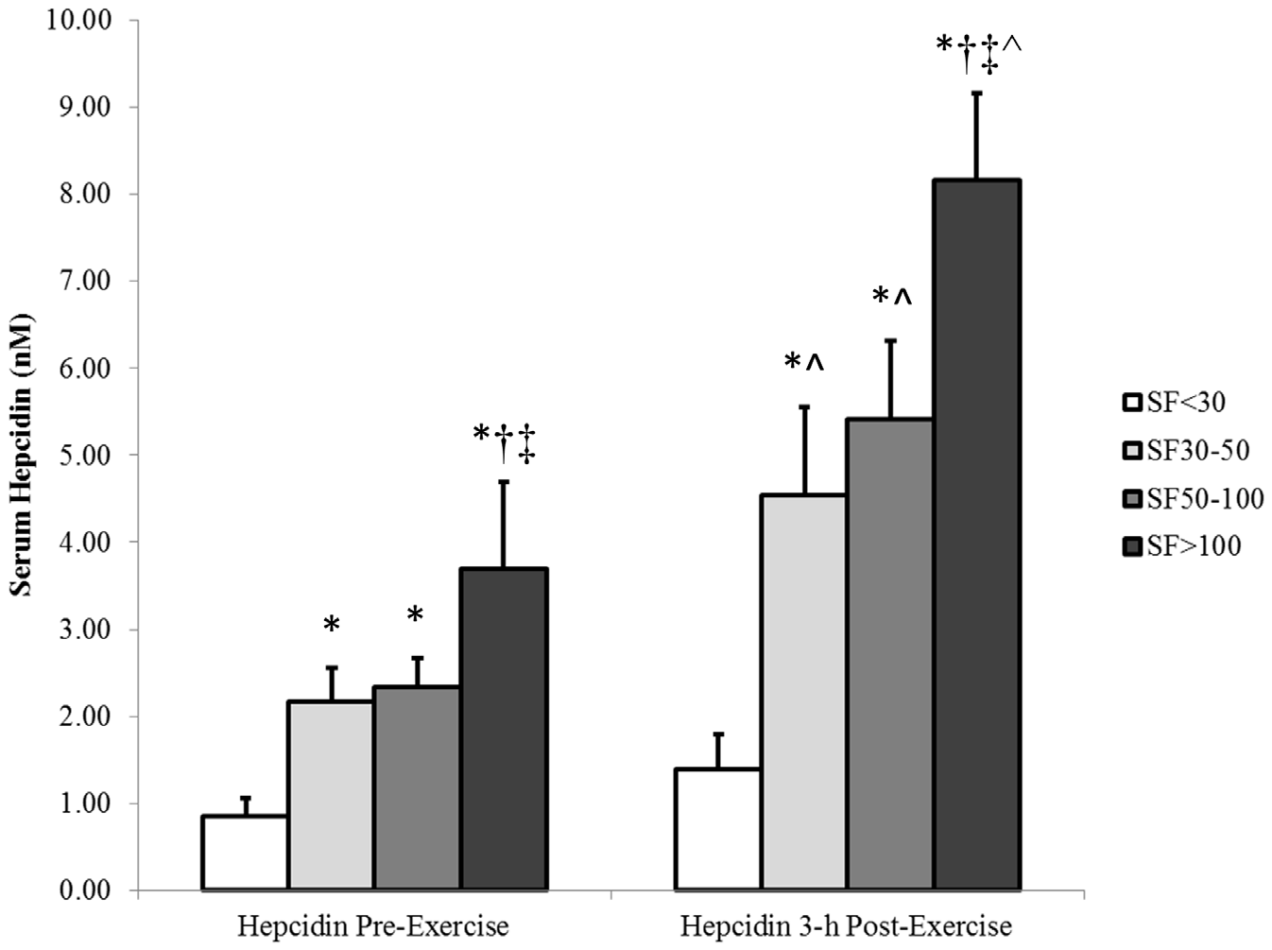
Mean (± SE) pre-exercise and 3 h post-exercise hepcidin-25 levels in athletes with baseline serum ferritin levels <30 μg/L (SF<30), between 30–50 μg/L (SF30–50), between 50–100 μg/L (SF50–100), and >100 μg/L (SF>100). * Significantly greater than SF<30. † Significantly greater than SF30–50. ‡ Significantly greater than SF50–100. ∧ Significantly greater than pre-exercise.

The pre- and 3 h post-exercise hepcidin-25 response is shown in Figure 1. Significant between group differences were evident for both the pre- (p = 0.01) and 3 h post-exercise (p = 0.01) levels, Specifically, the pre-exercise hepcidin-25 level was sequentially greater as the groups baseline serum ferritin levels increased (SF<30 vs. SF30–50: p = 0.03; SF<30 vs. SF50–100: p = 0.01; SF<30 vs. SF>100: p = 0.01; SF30–50 vs. SF>100: p = 0.01; SF50–100 vs. SF>100: p = 0.01); however, this effect was not evident when comparing SF30–50 to SF50–100 (p = 0.80). Furthermore, the same response was evident 3 h post-exercise (SF<30 vs. SF30–50: p = 0.04; SF<30 vs. SF50–100: p = 0.01; SF<30 vs. SF>100: p = 0.01; SF30–50 vs. SF>100: p = 0.01; SF50–100 vs. SF>100: p = 0.03), again with the effect not evident when comparing SF30–50 to SF50–100 (p = 0.57). Interestingly, the post-exercise hepcidin-25 levels were significantly greater than baseline in SF30–50 (p = 0.03), SF50–100 (p = 0.01), and SF>100 (p = 0.01); however, this response was not evident (p = 0.10) in the SF<30.

**Figure 2:**
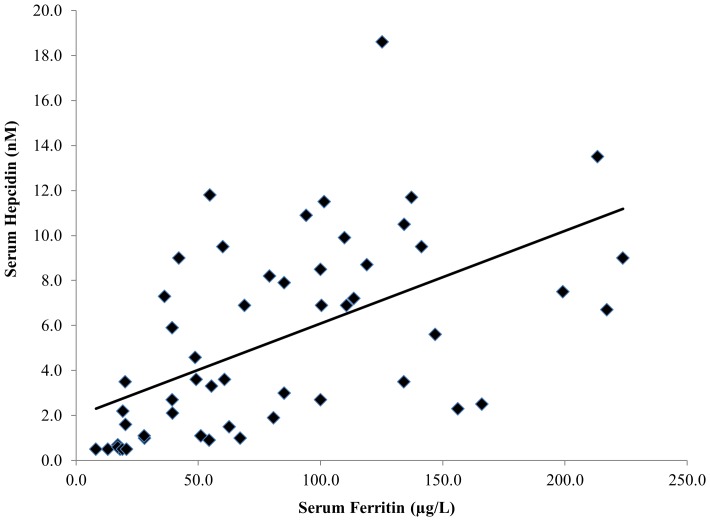
Scatter plot and linear trend line of each individual's 3 h post-exercise hepcidin response in association with their baseline serum ferritin levels (r = 0.52; p = 0.01).

Baseline serum ferritin levels showed a strong positive association to both the pre-exercise (r = 0.50; p = 0.01), and post-exercise (r = 0.52; p = 0.01) hepcidin-25 levels. Figure 2 shows the baseline serum ferritin and post-exercise hepcidin-25 association, in addition to the individual athlete response. A similar association was also seen between the post-exercise serum ferritin levels and post-exercise hepcidin-25 (r = 0.53; p = 0.01). There were no relationships found between measures of pre- or post-exercise IL-6 or serum iron with baseline or post-exercise hepcidin-25.

## Discussion

The results of this investigation showed that an athlete's iron status may dictate both the pre-exercise levels of hepcidin, and also the magnitude of hepcidin response to an acute exercise stimulus. Additionally, although a significant and comparable post-exercise IL-6 (suggesting an inflammatory response) and serum iron (suggesting a haemolytic stimulus) increase was evident between groups, it would seem that low serum ferritin levels (and the lack of post-exercise increase in this iron marker) were a greater stimulus for the suppression of post-exercise hepcidin activity, overriding the inflammatory-driven increases in hepcidin that were evident in the SF30–50, SF50–100, and SF>100 groups. However, it was also apparent that the increases to hepcidin levels were greatest in the SF>100 group, further implying that the body regulates this response relative to iron stores, with subtle reductions in the hormone levels as the serum ferritin levels fall, until switching the process off when the athlete reaches a point of compromised iron status.

Supressed baseline hepcidin levels in iron deficient populations comparative to healthy controls have been previously shown, where iron deficient rodents were maintained on an iron deplete diet (3 mg/kg wet weight), or healthy controls on an iron replete diet (159 mg/kg wet weight) for 6 weeks, whilst measuring the levels of hepcidin gene expression both before, and after treatment with lipopolysaccharide to induce inflammation [20]. These authors showed that the baseline hepcidin levels in the iron deficient group were significantly lower than those with normal iron stores. Furthermore, strong positive correlations between baseline serum ferritin and hepcidin levels amongst various populations of healthy individuals, and/or patients showing inflammatory disorders or iron overload have previously been shown [21]–[23]. As a result, the lower baseline levels of hepcidin reported here in the iron deficient group are not uncommon; however, the sequential increase in the resting levels of this hormone in relation to the baseline iron stores in an athletic population is novel, and suggests that the body's underlying mechanism controlling the absorption and recycling of iron responds appropriately at rest in the absence of an acute-phase inflammatory stimulus such as exercise.

When considering the post-exercise responses seen in this study however, it was evident that all four groups incurred a significant inflammatory stimulus from the exercise task. Subsequently, it appeared that the hepcidin levels were again sequentially higher as the baseline iron status of the athletes increased. Of interest however, is the fact that the elevations in hepcidin levels were not present in the iron deficient group. Increased hepcidin levels in response to an inflammatory stimulus has precedence in previous research reporting on unhealthy patients with anaemia of inflammation, who showed significantly greater circulating hepcidin levels compared to those of healthy controls and to individuals with iron deficiency [22]. The same differences were also reported between rheumatoid arthritis patients with inflammatory associated anaemia of chronic disease and those with iron deficiency [24]. Furthermore, when rodents were treated with lipopolysaccharide to induce inflammation, it was shown that the subsequent increase in hepcidin gene expression was significantly lower in an iron deficient group compared to healthy controls [20]. These authors suggested that although the inflammatory response invoked in their study was a strong inducer of hepcidin expression, chronic iron deficiency seemed able to counteract this effect. Such conclusions are reinforced by the findings that hepcidin levels in iron deficient and anaemic children with elevated inflammatory markers were significantly lower than those in non-anaemic children presenting with the same inflammatory state [25]. These authors concluded that the inflammatory-mediated stimuli are overridden by iron demand and erythropoiesis, which in turn, down regulates hepcidin synthesis [25]. The culmination of these previous findings in non-athletic populations provides justification for the sequentially higher pre- and post-exercise hepcidin levels seen here as the baseline iron status of the athletes increased, and for the non-response of the SF<30 group.

Similar to the investigations of populations presenting with inflammation from chronic disease, numerous exercise trials have shown significant post-exercise increases to circulating hepcidin levels in response to the inflammatory-driven increases in IL-6 [3], [4], [26]. However, the results from previous exercise-based studies have sporadically reported cases of hepcidin non-responders within the populations sampled [2], [3], with the suggestion that these participants may have been iron deficient. More recent work has supported this observation in longer-term training interventions, with lower baseline hepcidin levels reported in association with low serum ferritin levels [12], [13]. Regardless, in each of these past studies, the total number of confirmed iron deficient participants has either been very low [3], the iron status of the athletes was not measured [2], or the influence of these baseline measures was not considered in association with the acute post-exercise response [12], [13]. As a result, the current investigation highlights the post-exercise hepcidin response of a larger iron deficient athletic population, confirming that low baseline iron stores could explain the hepcidin non-responders seen in this previous research.

The post-exercise hepcidin responses reported here imply a sequential increase in activity positively associated with the baseline iron status of the athlete, irrespective of the inflammatory stimulus created by the exercise task. Again, this outcome suggests that hepcidin is responding to maintain iron homeostasis, sensing the need for increased dietary iron absorption as the serum ferritin levels of the athlete decrease. However, based on recent literature investigating the iron status of well-trained athletes [18], the baseline iron stores of the SF30–50 group (mean: 43.1 μg/L) can be considered ‘sub-optimal’; yet a post-exercise hepcidin response was still evident after 3 h of recovery in this group, which is of similar magnitude to those athletes presenting with healthy iron stores. Previously, an increase in hepcidin response by females presenting with sub-optimal (mean serum ferritin: 42 μg/L) iron stores has been shown [23]. These authors showed an increase in hepcidin levels as a result of greater iron availability through oral iron administration, suggesting that hepcidin suppression in individuals with low serum ferritin levels is attenuated, and that hepcidin concentrations may still increase despite low body iron stores. Unfortunately, the 3 h post-exercise time frame for hepcidin elevations seen here may coincide with an athlete's meal consumption that contains the majority of their dietary iron intake following a training session. As a result, it is possible that the post-exercise elevations in hepcidin activity are of most concern to athletes that present with a ‘sub-optimal’ iron status (i.e. those with serum ferritin levels ∼30–50 μg/L), since it is in these athletes where a significant post-exercise elevation in hepcidin will still occur, potentially reducing their ability to recycle and absorb iron from the diet, thereby increasing the likelihood that an iron deficiency may develop over time.

Although novel and interesting, it must be acknowledged that the exercise stimulus in this investigation was not standardised between all participants. However, all exercise trials were represented across the four serum ferritin groups, with the statistical analysis suggesting no significant differences for exercise duration or intensity between trials. Additionally, it should also be considered that the universal cut-off for defining an iron deficiency in athletes remains elusive. Regardless, the serum ferritin groups established here are based on recent literature [18], [19], and fit within the scope of previous work related to athletic-induced iron deficiency [1]–[7], [12], [18]. Finally, it should also be acknowledged that the influence of gender on this acute post-exercise response is not well understood. However, as a level of control, it should be noted that in each of our investigations, female athletes were only tested in the follicular phase of their menstrual cycle. Regardless, due to the influence that baseline serum ferritin has been shown to play here, a larger sample size of both genders with similar baseline serum ferritin levels would be required to investigate any gender differences appropriately, and as such, cannot be completed from the current data set. To this end, the results presented here should be interpreted with these considerations in mind, setting the scene for future research to further explore the influence of exercise intensity and duration, the cut-offs for iron status, and the gender effects on the haematological responses in athletes with varying degrees of iron status.

## Conclusion

In summary, applied sports physicians, dietitians and physiologists working with iron deficient athletes should continue to focus on increasing iron stores through food choices and oral supplementation; or via intramuscular and intravenous iron injections (in conjunction with attaining any appropriate Therapeutic Use Exemptions from governing anti-doping agencies). Furthermore, the training load and impact nature of the exercise prescribed to such athletes also needs to be considered, taking into account the potential influence of hemolysis on iron stores from different exercise modalities [4], [27]. However, if working with athletes that present with a ‘sub-optimal’ iron status (serum ferritin 30–50 μg/L), the focus should not only be on the supplementation of iron sources, but also on the timing of this supplementation, in order to avoid the peak periods of hepcidin elevation post-exercise.
